# Closed genome sequence of an *Arthrobacter globiformis* phage, SilentRX

**DOI:** 10.1128/mra.00273-25

**Published:** 2025-09-12

**Authors:** Ashna Siddiqui, Raunak Vijay, Ethan G. Edwards, Meliha Ulker, Fardeen A. Siddiqui, Thomas J. Gerton, Rachel E. Anastasi, Dylan J. Conroy, Isabelle E. Laizure, Joshua D. Reynolds, Kelsie Duggan, Kristen C Johnson, Kyle S. MacLea

**Affiliations:** 1 Biotechnology Program, University of New Hampshire 41567, Manchester, New Hampshire, USA; 2 Graduate Program in Biotechnology: Industrial and Biomedical Sciences, University of New Hampshire 41567, Manchester, New Hampshire, USA; 3 Biology Program, University of New Hampshire 41567, Manchester, New Hampshire, USA; 4 Department of Life Sciences, University of New Hampshire 41567, Manchester, New Hampshire, USA; Portland State University, Portland, Oregon, USA

**Keywords:** actinobacteria, soil, bacteriophage, SEA-PHAGES, lytic, siphoviridae

## Abstract

We sequenced the complete genome of SilentRX, an actinobacteriophage with siphovirus morphology in cluster AP that infects *Arthrobacter globiformis* NRRL B-2979. The phage possessed a capsid width of 55 nm and a tail length of 215 nm. With a length of 65,232 bp, the genome contained 102 predicted protein-coding genes.

## ANNOUNCEMENT

**Fig 1:**
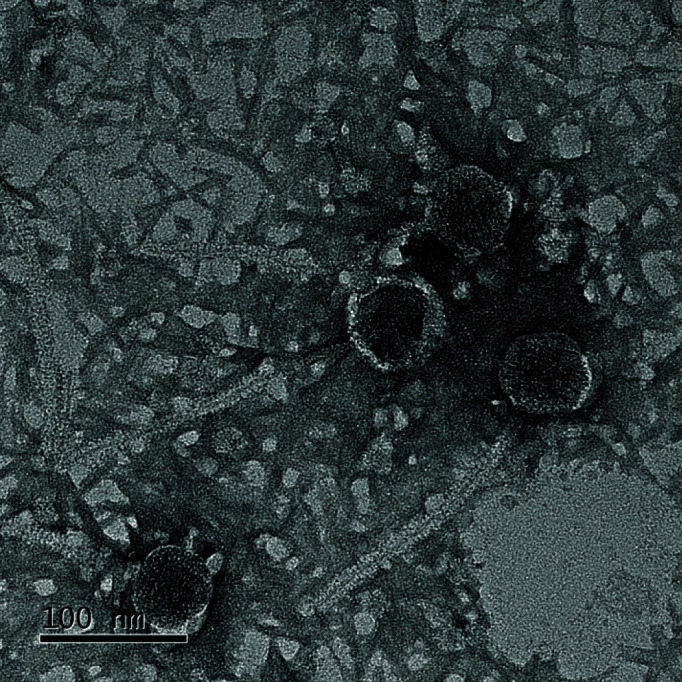
Transmission electron micrograph of *Arthrobacter globiformis* phage SilentRX. Transmission electron microscopy (TEM) imaging of phage SilentRX shows a siphoviral morphology including a 55 nm capsid head and 215 nm siphoviral non-contractile tail. Scale bar = 100 nm.

SilentRX is a lytic actinobacteriophage that infects *Arthrobacter globiformis* NRRL B-2979. It was originally isolated from sandy and slightly moist soil in Manchester, NH, USA (coordinates: 42.994046°N, 71.512123°W) in 2019. SilentRX was isolated, purified, and amplified using SEA-PHAGES direct isolation protocols (1). SilentRX was grown on double-layer peptone-yeast-calcium agar supplemented with 1 mM calcium chloride, 10 µg/mL cycloheximide, and 40% dextrose. The phage formed 0.2–1.2 mm circular plaques, which were clear-centered with surrounding turbidity, on bacterial lawns grown at 26°C. A high-titer lysate of SilentRX was prepared as previously described (2) and a single titer measurement was estimated at 1.11 × 10^10^ pfu/mL. Lysate was deposited on PELCO carbon conductive tabs (16084-1; Ted Pella, Redding, CA, USA) and negatively stained with 1% uranyl acetate, following established procedures (3, 4). Transmission electron microscopy (TEM) analysis using a Tecnai F20 microscope (Dartmouth Electron Microscope Facility, Hanover, NH, USA) revealed that SilentRX had a siphovirus morphology, and its capsid head and noncontractile tail measured 55 nm in diameter and 215 nm in length, respectively (Fig. 1).

DNA was extracted from high-titer lysate using the Wizard DNA Clean-Up Kit (A7280, Promega, Madison, WI, USA). A sequencing library prepared for SilentRX using the Ultra II FS kit (E7805S, NEB, Ipswich, MA, USA) was sequenced on Illumina MiSeq v3 and yielded 439.2k 100 bp single-end reads. Assembly of reads with Newbler v2.9 (Roche) resulted in 301-fold genome coverage. SilentRX was assigned to cluster AP, found to have direct terminal repeat ends, and was determined to be completely closed as described (5).

SilentRX’s genome was annotated utilizing DNA Master v5.23.3 (https://phagesdb.org/DNAMaster/) (6). Coding sequences were predicted and functionally annotated using GLIMMER v3.02 (7), GeneMarkS v2.0 (8), Phamerator (9), Starterator v1.0.1 (https://github.com/SEA-PHAGES/starterator), BLAST (10), and HHpred (11). All programs used default settings. One hundred two putative protein-coding genes were identified in SilentRX. No tRNA genes were found using ARAGORN v.1.2.41 or tRNAscan-SE v.2 (12, 13).

SilentRX has a genome length of 65,232 bp. Out of 102 predicted protein-coding genes, 40 are transcribed in the forward direction, 62 are transcribed in the reverse direction, 29 (28%) have assigned functions, and 10 have no known homologs in any phage genome. SilentRX has genes that resemble those of bacteriophages with siphoviral morphology, including portal protein, terminase, head-to-tail adaptor (14), and the lytic protein endolysin (15). Furthermore, there is no evidence of a temperate lifestyle since it lacks obvious genes for lysogeny (16).

Bacteriophages typically exhibit an equal or reduced GC content percentage compared to their hosts (17–19). A higher GC content of a phage genome or gene could be evidence of horizontal gene transfer (16, 17). SilentRX has an average GC content of 67.12% and *A. globiformis* NRRL B-2979 has an average GC content of 66.2%. Gene 13 of SilentRX, a putative DNA-binding protein, has a higher GC content (73.54%). This could be the result of foreign, horizontally transferred DNA. SilentRX shows genetic diversity, including orphan homologs and GC shifts, suggesting gene acquisition from other sources.

## Data Availability

The genome sequence of bacteriophage SilentRX has been deposited in DDBJ/ENA/GenBank under accession number MW862992. The raw Illumina data from BioSample SAMN39610766 were submitted to the NCBI Sequence Read Archive (SRA) under experiment accession number SRX23452937.

## References

[1] Hatfull GF. 2015. Innovations in undergraduate science education: going viral. J Virol 89:8111–8113. doi:10.1128/JVI.03003-14 26018168 PMC4524241

[2] Poxleitner M, Pope W, Jacobs-Sera D, Sivanathan V, Hatfull G. 2018. HHMI SEA-PHAGES phage discovery guide. Howard Hughes Medical Institute, Chevy Chase. Available from: https://seaphagesphagediscoveryguide.helpdocsonline.com

[3] Ackermann HW. 2009. Basic Phage Electron Microscopy, p 113–126. In Clokie MRJ, Kropinski AM (ed), Bacteriophages. Humana Press, Totowa, NJ. 10.1007/978-1-60327-164-6_12 19066816

[4] Jordan TC, Burnett SH, Carson S, Caruso SM, Clase K, DeJong RJ, Dennehy JJ, Denver DR, Dunbar D, Elgin SCR, et al.. 2014. A broadly implementable research course in phage discovery and genomics for first-year undergraduate students. mBio 5:e01051-13. doi:10.1128/mBio.01051-13 24496795 PMC3950523

[5] Russell DA. 2018. Sequencing, assembling, and finishing complete bacteriophage genomes. Methods Mol Biol 1681:109–125. doi:10.1007/978-1-4939-7343-9_9 29134591

[6] Pope WH, Jacobs-Sera D. 2018. Annotation of bacteriophage genome sequences using DNA Master: an overview. Methods Mol Biol 1681:217–229. doi:10.1007/978-1-4939-7343-9_16 29134598

[7] Delcher AL, Harmon D, Kasif S, White O, Salzberg SL. 1999. Improved microbial gene identification with GLIMMER. Nucleic Acids Res 27:4636–4641. doi:10.1093/nar/27.23.4636 10556321 PMC148753

[8] Besemer J, Borodovsky M. 2005. GeneMark: web software for gene finding in prokaryotes, eukaryotes and viruses. Nucleic Acids Res 33:W451–4. doi:10.1093/nar/gki487 15980510 PMC1160247

[9] Cresawn SG, Bogel M, Day N, Jacobs-Sera D, Hendrix RW, Hatfull GF. 2011. Phamerator: a bioinformatic tool for comparative bacteriophage genomics. BMC Bioinformatics 12:395. doi:10.1186/1471-2105-12-395 21991981 PMC3233612

[10] Altschul SF, Gish W, Miller W, Myers EW, Lipman DJ. 1990. Basic local alignment search tool. J Mol Biol 215:403–410. doi:10.1016/S0022-2836(05)80360-2 2231712

[11] Söding J, Biegert A, Lupas AN. 2005. The HHpred interactive server for protein homology detection and structure prediction. Nucleic Acids Res 33:W244–8. doi:10.1093/nar/gki408 15980461 PMC1160169

[12] Laslett D, Canback B. 2004. ARAGORN, a program to detect tRNA genes and tmRNA genes in nucleotide sequences. Nucleic Acids Res 32:11–16. doi:10.1093/nar/gkh152 14704338 PMC373265

[13] Chan PP, Lowe TM. 2019. tRNAscan-SE: searching for tRNA genes in genomic sequences. Methods Mol Biol 1962:1–14. doi:10.1007/978-1-4939-9173-0_1 31020551 PMC6768409

[14] Klyczek KK, Bonilla JA, Jacobs-Sera D, Adair TL, Afram P, Allen KG, Archambault ML, Aziz RM, Bagnasco FG, Ball SL, et al.. 2017. Tales of diversity: genomic and morphological characteristics of forty-six Arthrobacter phages. Plos One 12:e0180517. doi:10.1371/journal.pone.0180517 28715480 PMC5513430

[15] Shi Y, Li N, Yan Y, Wang H, Li Y, Lu C, Sun J. 2012. Combined antibacterial activity of phage lytic proteins holin and lysin from Streptococcus suis bacteriophage SMP. Curr Microbiol 65:28–34. doi:10.1007/s00284-012-0119-2 22526567

[16] Demo S, Kapinos A, Bernardino A, Guardino K, Hobbs B, Hoh K, Lee E, Vuong I, Reddi K, Freise AC, Moberg Parker J. 2021. BlueFeather, the singleton that wasn’t: shared gene content analysis supports expansion of Arthrobacter phage Cluster FE. PLoS One 16:e0248418. doi:10.1371/journal.pone.0248418 33711060 PMC7954295

[17] Ravenhall M, Škunca N, Lassalle F, Dessimoz C. 2015. Inferring horizontal gene transfer. Plos Comput Biol 11:e1004095. doi:10.1371/journal.pcbi.1004095 26020646 PMC4462595

[18] Bohlin J, Pettersson JO. 2019. Evolution of genomic base composition: from single cell microbes to multicellular animals. Comput Struct Biotechnol J 17:362–370. doi:10.1016/j.csbj.2019.03.001 30949307 PMC6429543

[19] de Melo ACC, da Mata Gomes A, Melo FL, Ardisson-Araújo DMP, de Vargas APC, Ely VL, Kitajima EW, Ribeiro BM, Wolff JLC. 2019. Characterization of a bacteriophage with broad host range against strains of Pseudomonas aeruginosa isolated from domestic animals. BMC Microbiol 19:134. doi:10.1186/s12866-019-1481-z 31208333 PMC6580649

